# Roles of the integrated stress response in regulation of inflammatory reactions

**DOI:** 10.3389/fimmu.2026.1747401

**Published:** 2026-02-25

**Authors:** Fan Jiang, Guei-Sheung Liu, Junjun Liu, Xiaopei Cui, Yanqiu Xing

**Affiliations:** 1 Gerontology and Anti-Aging Research Laboratory, Department of Geriatric Medicine, Qilu Hospital of Shandong University, Jinan, Shandong, China; 2 Jinan Clinical Research Center for Geriatric Medicine, Qilu Hospital of Shandong University, Jinan, Shandong, China; 3 Centre for Eye Research Australia, Royal Victorian Eye and Ear Hospital, East Melbourne, VIC, Australia; 4 Ophthalmology, Department of Surgery, University of Melbourne, East Melbourne, VIC, Australia; 5 Menzies Institute for Medical Research, University of Tasmania, Hobart, TAS, Australia

**Keywords:** anti-inflammation, ATF4, eIF2a, GADD34, inflammatory disease, integrated stress response, phosphorylation, protein translation

## Abstract

The integrated stress response (ISR) is a conserved cyto-protective mechanism, which has fundamental roles in maintaining cell viability under various conditions when intracellular and/or extracellular homeostasis is disrupted. ISR features phosphorylation of the alpha subunit of eukaryotic translation initiation factor 2 (eIF2α), leading to a global reduction in protein synthesis. Emerging evidence suggests that activation of ISR may have anti-inflammatory effects. In this concise review, we summarize the current experimental evidence in this regard from both *in vitro* and *in vivo* studies. It is suggested that ISR may represent a potential drug target for developing novel anti-inflammatory therapies.

## 1 The integrated stress response

**Figure 1:**
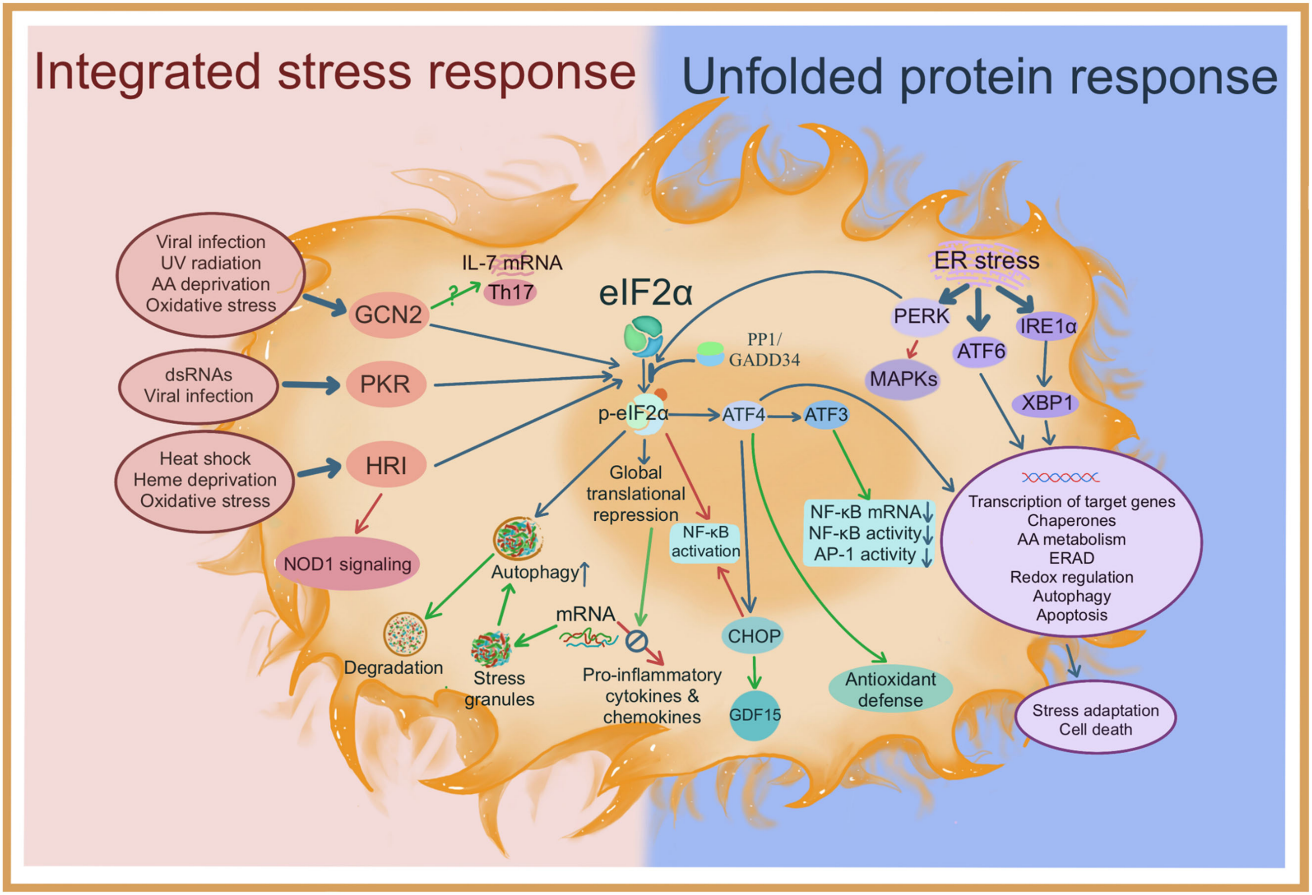
Signal transduction mechanisms of the integrated stress response and the unfolded protein response, and their interactions with inflammatory reactions. Potential anti-inflammatory effects are indicated by green arrows; possible pro-inflammatory effects are indicated by red arrows. NOD1, nucleotide binding oligomerization domain containing 1; MAPKs, mitogen-activated protein kinases; IRE1α, inositol-requiring transmembrane kinase endoribonuclease-1α; XBP1, X-box binding protein 1; AA, amino acid; ERAD, endoplasmic reticulum-associated degradation.

The integrated stress response (ISR) is an ancient cyto-protective mechanism found in eukaryotes, which is activated by various intrinsic and extrinsic stressor stimuli and results in a global reduction in protein synthesis (1–5). Timely shutting down of gene translation is vitally important for cell adaptation to stress conditions, since protein synthesis is one of the most sophisticated and resource-consuming biological processes in cells (1, 5, 6). Reduced protein translation can allow cells to conserve resources and initiate a reconfiguration of gene expression to effectively cope with stress conditions (1, 3). Cell-extrinsic stimuli of ISR include amino acid deprivation, glucose deprivation, heme deprivation, hypoxia, oxidative stress, UV irradiation, and viral infection (1, 3, 4). The most well-characterized cell-intrinsic stimulus of ISR is endoplasmic reticulum (ER) stress, which is caused by the accumulation of unfolded proteins in the ER (7). For this reason, there is a considerable overlap between the signaling mechanisms and outcomes of ISR and unfolded protein response (UPR), another important cellular stress response activated in the presence of ES stress (8) (**Figure 1**).

The pivotal signaling module in ISR is the phosphorylation of eukaryotic translation initiation factor 2 alpha subunit (eIF2α) on Ser51 (1–6). The translation initiation factor eIF2 (a heterotrimer of an α, β, and γ subunit) forms a ternary complex with GTP and Met-tRNAi (charged methionyl-initiator tRNA), which is instrumental in AUG-dependent translation initiation. At the AUG start codon, the GTP in the ternary complex is hydrolyzed to GDP, allowing the release of Met-tRNAi and the assembly of a functional ribosome, followed by commencement of the elongation phase. The dissociated eIF2-GDP is then converted to eIF2-GTP through the action of its guanine nucleotide exchange factor, eIF2B, allowing it to be engaged in a new round of translation initiation. This eIF2-GDP/eIF2-GTP recycling process is the rate-limiting step for ternary complex formation and subsequent AUG-initiated mRNA translation. Phosphorylation of eIF2α transforms it from an eIF2B substrate into an eIF2B inhibitor, leading to a reduction in the availability of eIF2-GTP, thereby limiting the rate of ternary complex formation. In a word, ISR-induced eIF2α phosphorylation represses protein synthesis by reducing the rate of ternary complex formation and subsequent translation initiation (1–4, 6).

In mammalian cells, phosphorylation of eIF2α is mediated by one of the four well-established eIF2α kinases, namely protein kinase R (PKR) (also known as protein kinase RNA-activated), PKR-like endoplasmic reticulum kinase (PERK), general control non-depressible 2 (GCN2), and heme regulated inhibitor (HRI) (3, 4). PKR is activated by double-stranded RNAs and hence participates in the innate immune response to viral infection. PERK is localized to the ER membrane, and is activated in response to ER stress. GCN2 is activated by amino acid deprivation. There is evidence showing that GCN2 can also be activated by other stresses such as ultraviolet radiation, viral infection, serum starvation, and oxidative stress. HRI is primarily activated upon heme deprivation and has a specialized role in erythroid cells dedicated to hemoglobin synthesis. On the other hand, this kinase is also responsive to multiple other cellular stresses, such as oxidative stress, heat shock, and cytosolic protein aggregation (2, 4, 6).

While ISR results in a global reduction in mRNA translation, a small group of mRNAs are preferentially translated during ISR, including those encoding activating transcription factor (ATF) 4, ATF5, C/EBP-homologous protein (CHOP), and growth arrest and DNA damage-inducible gene 34 (GADD34), through a mechanism known as “upstream open reading frame bypass” (4, 8). Specifically, these mRNAs contain short inhibitory upstream open reading frames that prevent initiation at their canonical AUG start codon under unstressed conditions. However, stress-induced eIF2α phosphorylation reduces the efficiency of ternary complex formation. As a result, the translation initiation at the upstream open reading frame is bypassed, enabling the scanning ribosomes to initiate at the downstream target open reading frame instead (4). Among the preferentially translated genes, ATF4 is the most well-characterized example, which encodes a basic leucine zipper transcription factor. ATF4 orchestrates a complex network of transcriptional reprogramming in ISR (9).

Timely termination of the ISR, which is important for restoration of the cellular homeostasis in protein synthesis upon removal of the stress condition, is a tightly regulated process. In mammalian cells, p-eIF2α dephosphorylation is mediated by protein phosphatase 1 (PP1) holoenzyme (10). PP1 is a heterodimeric Ser/Thr phosphatase comprising one of the 3 isoforms of the catalytic subunit (PP1α, PP1β, and PP1γ) (11), and one of the numerous PP1 regulatory subunits (also called PP1-interacting proteins) (12). It is recognized that the catalytic subunits of PP1 have little substrate selectivity, whereas selective substrate-targeting of the enzyme is primarily determined by the diversity of regulatory subunits (12). The specificity to p-eIF2α is achieved by two regulatory subunits, GADD34 (gene name PPP1R15A) (13) and CReP (constitutive repressor of eIF2α phosphorylation, gene name PPP1R15B) (14). While CReP is constitutively expressed and responsible for a slow but steady rate of p-eIF2α dephosphorylation, the expression of GADD34 is induced during ISR via ATF4-dependent transcriptional upregulation and upstream open reading frame-dependent preferential translation, providing a critical negative feedback mechanism (2, 10).

In general, ISR is thought to be an evolutionarily conserved cyto-protective intracellular signaling network, acting to aid the cell, tissue, and organism to adapt to harmful environmental changes and maintain health (15–19). Phosphorylation of eIF2α represents a critical tier of translational control, especially in stressed cells, in addition to other well-documented regulatory signaling mechanisms such as the mechanistic target of rapamycin/mTOR and mitogen-activated protein kinase/MAPK pathways (20). Interestingly, results from a series of recent studies suggest that activation of ISR may have significant roles in inhibiting inflammatory reactions in various cell types and in a few *in vivo* models. In this concise review, we summarize the emerging evidence in this regard.

## 2 Evidence in macrophages

In two separate studies, a research group used arsenite (21) or halofuginone (a small molecule derivative of the plant alkaloid febrifugine, which is extracted from *Dichroa febrifuga*) (22) as ISR inducers in a murine macrophage cell line and/or primary bone marrow-derived macrophages, and demonstrated that these treatments reduced interleukin (IL)-1β production induced by lipopolysaccharides (LPS). The authors excluded that the effects were due to altered transcription of the IL-1β gene; rather, the authors proposed that, at least partly, ISR activation shuttled the untranslated mRNAs of IL-1β to stress granules (21, 22), a ribonucleoprotein-based cellular compartment formed upon exposure to various stressors (23). Consistent with this notion, the authors provided evidence suggesting that the untranslated IL-1β mRNAs were eventually removed through autophagic clearance of the stress granules (21, 22). In agreement, eIF2α phosphorylation was also shown to have an important role in facilitating autophagy in both mammalian cells (24) and yeast (25). It was shown that the production of tumor necrosis factor (TNF)-α was similarly reduced by the treatments, whereas the response of IL-6 was variable. Nonetheless, it was not verified whether the same mechanism regulated the production of TNF-α. Moreover, either arsenite or halofuginone is non-specific for eIF2α kinases; hence, it was not surprising that the authors also observed some “off-target” effects of these compounds in these studies (*e.g.*, reduction of reactive oxygen species by halofuginone and inhibition of caspase-1-dependent cleavage of pro-IL-1β by arsenite) (21, 22).

We recently employed two strategies to target eIF2α in a more specific manner and investigated the effects of increased eIF2α phosphorylation on the heightened cytokine production in LPS-activated macrophages (mimicking a cytokine storm-like response). Firstly, eIF2α-S51D mutant was ectopically overexpressed to mimic the phosphorylated state of eIF2α; secondly, pharmacological treatment with salubrinal was applied to inhibit eIF2α dephosphorylation (26). Although the specificity of salubrinal as a PP1/GADD34 inhibitor is questioned based on some experimental evidence (10, 13), the enhancing effect of salubrinal on eIF2α phosphorylation has been well documented (10), and was confirmed in our own study (26). In addition, these experimental approaches bypassed the initial induction of cellular stresses, which may have potential eIF2α-independent, counter-balancing effects on inflammation [see references (27) and (28)]. The results demonstrated that in the RAW264.7 macrophage cell line, eIF2α-S51D, salubrinal, and GADD34 gene silencing all significantly inhibited the production of TNF-α, pro-IL-1β, and IL-6, without changing their mRNA levels. Further analyses supported the conclusion that these anti-inflammatory effects were mediated by decoupling of the gene transcription and protein translation. We also excluded the involvement of changes in the Toll-like receptor 4 (TLR4) or mammalian target of rapamycin (mTOR) signaling. These data suggest that timely activation of ISR by modulating eIF2α phosphorylation may act to brake the life-threatening cytokine production (cytokine storm-like response) during uncontrolled macrophage activation, such as the systemic inflammatory response syndrome found in bacterial sepsis.

Upon viral infection, host cells promptly initiate a complicated anti-viral program. On one hand, the invasion of pathogens is detected by various pattern recognition receptors (29, 30), leading to increased expression of type I interferons (IFNs). IFNs orchestrate the expression of a myriad of target genes with multiple anti-viral functions (including PKR) (31). On the other hand, viral infection triggers ISR (32, 33). Viral double-stranded RNAs in the cytosol activate PKR; viral proteins in the ER may activate PERK; in addition, GCN2 may be activated by binding to viral RNA genomes (32) and by virus-induced amino acid imbalance (34). Global translational repression may restrict replication of the virus since viruses depend on the host translation apparatus to express viral proteins. In parallel, formation of stress granules is proposed to be able to disrupt virus replication by sequestration of viral factors and/or by acting as a signaling platform to coordinate the expression of anti-viral genes (32, 33). Nevertheless, at present, it is not clearly understood how cells balance the translation of the anti-viral genes in the presence of global translational repression during viral infection (32). In RAW264.7 cells, Brocard and colleagues showed that murine norovirus infection initiated an amino acid starvation-like response via GCN2 (34); this stress condition subsequently triggered an anti-inflammatory transcriptomic program, which was mediated by ATF3, a stress-inducible transcription factor with profound anti-inflammatory functions (35). Moreover, activation of GCN2 in norovirus-infected macrophages upregulated the transcription of GDF15 (growth differentiation factor 15), a cytokine of the transforming growth factor-β superfamily. The transcription of GDF15 is controlled by CHOP (36), a basic leucine zipper transcription factor that is induced during ISR and UPR (see **Figure 1**). Interestingly, our recent study indicated that GDF15 had an anti-inflammatory role in macrophages (37). Uniquely, the amino acid starvation-like response induced by norovirus infection was not accompanied by general p-eIF2α-dependent translational repression, arguing that the final effects of the anti-inflammatory transcriptomic program would not be interfered with at the translational level (34). It should be noted that the anti-inflammatory property of GCN2-ATF3 signaling during norovirus infection may be disadvantageous for host defenses, since appropriate production of pro-inflammatory cytokines is required for normal antiviral immunity. Nevertheless, these results raise a possibility that ISR-related ATF3 signaling may constitute an additional layer of regulation of pro-inflammatory reaction in macrophages.

## 3 Evidence in T lymphocytes

Recently, Asada et al. discovered that in a subset of CD4^+^ memory T cells, ISR signaling (eIF2α phosphorylation) was maintained at a relatively high level at steady state (38). In unstimulated cells, mRNAs encoding pro-inflammatory cytokines (such as IFN-γ, TNF-α, IL-17, and granulocyte–macrophage colony-stimulating factor) were expressed and stored with minimal protein production. Upon stimulation with phorbol ester plus ionomycin, these cells exhibited prompt eIF2α dephosphorylation and unleashed translation of the stored cytokine mRNAs. It was estimated that under the resting condition, around 20% of the cytokine mRNA was under translation, while the fraction increased to 80% after T cell activation. Consistently, the authors demonstrated that the increased translation of the cytokines in stimulated cells could be reversed by reinforcing eIF2α phosphorylation with arsenite or Sal003 [a derivative of salubrinal, a potent and cell-permeable inhibitor of eIF2α dephosphorylation (39)], or with eIF2α-S51D overexpression (38). These results are consistent with those observed in macrophages (26), clearly demonstrating that the canonical ISR negatively regulates pro-inflammatory reactions in immune cells via translational repression (38).

T helper 17 (Th17) cells, characterized by production of IL-17, are implicated in the pathogenesis of various inflammatory and autoimmune diseases. Sundrud et al. demonstrated that treatment with halofuginone selectively inhibited the differentiation of T cells into Th17 and suppressed the mRNA expression of IL-17, while the differentiation into Th1 or Th2 cells was not affected (40). This effect was attributable to ISR induction, featuring increases in eIF2α phosphorylation and ATF4 protein expression, through a GCN2-mediated amino acid starvation-like response, as evidenced by its mimicry by selected amino acid restriction, reversal by supplementation of excess free amino acids, and abrogation by GCN2 gene silencing. Importantly, the authors showed that halofuginone treatment inhibited Th17 response *in vivo* and ameliorated autoimmune inflammation in a mouse model of experimental autoimmune encephalomyelitis (40). This group further explored the molecular mechanism by which halofuginone induced the amino acid starvation-like response, showing that halofuginone could inhibit the enzyme glutamyl-prolyl tRNA synthetase by competing with the proline-binding site, causing the accumulation of uncharged proline-tRNA, thereby mimicking a condition of reduced cellular proline availability (41). However, it remains unclear how halofuginone-induced ISR modulates the mRNA level of IL-17 and whether halofuginone alters the translation of IL-17 protein. Interestingly, a recent study by another group confirmed that halofuginone could trigger GCN2-dependent eIF2α phosphorylation, which was abrogated by proline supplementation; however, the attenuation of protein synthesis in response to halofuginone appeared to be independent of the GCN2-eIF2α pathway, but might be related to defects in translation elongation (42).

## 4 Evidence in fibroblasts

In primary periodontal ligament fibroblasts stimulated with LPS, Shen et al. showed that induction of UPR with tunicamycin or thapsigargin reduced both mRNA and protein expressions of IL-1β, IL-6, TNF-α, and IL-8 (43). However, the mechanisms underlying these anti-inflammatory effects of UPR induction were elusive. The authors showed that tunicamycin or thapsigargin also attenuated TLR4 protein expression and the nuclear factor (NF)-κB signaling, which might explain the decreased transcription of the cytokine genes; however, it was not clear whether p-eIF2α-dependent translational regulation had any role in causing the reduced cytokine production. It is noted that UPR inducers may regulate cytokine gene translation independent of p-eIF2α. For example, in our previous study, we found that pre-treatment with thapsigargin reduced the production of pro-inflammatory cytokines in LPS-activated macrophages by inhibiting the activity of mTOR (44).

More data were obtained recently by Payea and colleagues in a study carried out in the human fibroblast cell line IMR-90 (45). It was shown that induction of cellular senescence in fibroblasts increased eIF2α phosphorylation to a level comparable to that in thapsigargin-treated cells; despite this, however, senescent cells failed to initiate the canonical ISR because of diminished protein expression of ATF4. Presumably, the lack of ATF4 response to eIF2α phosphorylation in senescent cells was due to an increase in the threshold of responsiveness of the upstream open reading frame bypass mechanism that governs ATF4 translation (45). In parallel, it was found that under the thapsigargin-induced stress condition, senescent cells specifically upregulated a number of mRNAs encoding proteins implicated in inflammatory pathways, a response that was reversed by re-expression of ATF4 protein. These data suggest that stress-induced ATF4 expression may intrinsically mediate an anti-inflammatory transcriptional signature, at least under the specified experimental conditions. However, it is unclear how senescent cells reconcile the increased transcription with the global reduction in protein translation.

## 5 *In vivo* evidence

In a murine model of autoimmune glomerulonephritis, it was demonstrated that treatment with Raphin1, a selective inhibitor of PPP1R15B (46), significantly increased the level of p-eIF2α in kidney tissue-resident memory T cells and reduced glomerular crescent formation and albuminuria (38). This evidence supports a therapeutic effect of targeting the ISR-eIF2α pathway on immune-mediated tissue inflammation and damage. Recently, we showed that administration of salubrinal to block p-eIF2α dephosphorylation exhibited striking protective effects against the development of acute lung injury induced by endotoxemia in mouse models (26). These effects included reduced leukocyte infiltrations in both the interstitial and intra-alveolar spaces, reduced edema of the alveolar wall, and reduced airway congestion. Moreover, salubrinal treatment alleviated inflammation-induced damage to the vascular endothelial barrier function (26). In the lungs, salubrinal significantly reduced the protein levels of TNF-α, IL-6, and IL-1β; in contrast, salubrinal caused moderate increases in the mRNA level of these cytokines, supporting the notion that the anti-inflammatory effects of salubrinal were related to ISR-induced translational repression.

GCN2 is not required for maintaining tissue homeostasis in the normal intestine; however, deletion of the GCN2 gene in antigen-presenting cells or intestinal epithelial cells resulted in increased prevalence of Th17 cells, inflammasome activation, and enhanced IL-1β production in a murine model of inflammatory bowel disease (47). Interestingly, it was shown that the anti-inflammatory action of GCN2 in the gut was only partly dependent on eIF2α; rather, GCN2 produced the effects mainly by promoting autophagy and antioxidant defense (47). Importantly, the authors demonstrated that activating the GCN2 signaling by dietary amino acid restriction exhibited therapeutic benefits against intestinal inflammation (47).

Increased inflammatory reactions in metabolic organs/tissues, such as the white adipose tissue, pancreatic islets, and the liver, are tightly involved in the pathogenesis and complications of type 2 diabetes (48, 49). In a mouse model of diet-induced obesity, induction of GCN2-mediated ISR by oral administration of halofuginone caused multiple metabolic benefits, including improved glucose tolerance, reduced weight gain, decreased insulin resistance, and decreased serum insulin level (50). Conversely, inhibiting PERK with the small molecule inhibitor GSK2656157 aggravated the diabetic phenotype. These data indicate a possible therapeutic benefit of enhancing ISR signaling by activating GCN2 for treating obesity-related diabetes (50). Unfortunately, no inflammatory markers were measured in this study.

## 6 Does ISR have pro-inflammatory effects?

Literature research identified numerous reports that linked ER stress to inflammation. However, most of these used eIF2α phosphorylation as a marker of the UPR (see **Figure 1**), rather than attempting to establish a causal relationship between eIF2α and inflammation. More direct evidence has been obtained from several studies showing that eIF2α phosphorylation can activate NF-κB (27, 28), which drives the transcription of multiple pro-inflammatory factors. This response seems to involve multiple mechanisms (51–53). Nevertheless, the transcriptional activation effects mediated by NF-κB may be dampened to some degree by the concomitant global reduction in mRNA translation. Supporting this argument, our study demonstrated that treatment of endotoxemic mice with salubrinal tended to increase the mRNA levels of TNF-α, IL-6, and IL-1β, whereas the protein levels of these cytokines were reduced (26).

In mouse embryonic fibroblasts, researchers showed that the eIF2α kinase HRI was involved in facilitating the assembly of NOD1 signalosome via p-eIF2α-ATF4-dependent expression of heat shock protein HSPB8 (54). NOD1 is an intracellular pattern recognition receptor for the detection of bacterial infection, which promotes inflammatory cytokine production by activating NF-κB and mitogen-activated protein kinases (55). The ATF4 target gene CHOP was shown to facilitate NF-κB activation and chemokine expression in pancreatic β-cells (56). Moreover, PERK activation can promote IL-6 and IL-8 production via increased activation of mitogen-activated protein kinases p38 and/or ERK (28).

Plenty of studies reported a pro-inflammatory role of ATF4 in different *in vitro* and *in vivo* models. However, several lines of evidence indicate that ATF4 may also orchestrate an anti-inflammatory transcriptional signature, at least under specific experimental settings. For example, as mentioned above, it was shown that failure of ATF4 expression in stressed senescent cells was associated with upregulated transcription of multiple genes with pro-inflammatory functions (45). The potential anti-inflammatory function of ATF4 seems to be partly related to enhanced antioxidant defense (27), whereas ATF4 does not directly stimulate the transcription of inflammation-inhibiting factors (9). Oxidative stress is a well-recognized contributor to the development and perpetuation of inflammation (57). ATF4-deficient cells can only grow in culture with supplemental antioxidant substances, and withdrawal of the antioxidants initiates a rapid increase in intracellular reactive oxygen species followed by cell death (15). ATF4 may elicit enhanced antioxidant defense by (1) activating genes involved in the import and metabolism of thiol-containing amino acids (principally cysteine) (15); (2) stimulating the expression of Nrf2 (nuclear factor erythroid 2-related factor 2), a master transcription factor that orchestrates the expression of multiple antioxidant and phase II detoxification enzymes, thereby maintaining cellular redox homeostasis (58); (3) in a cell-specific manner, promoting expression of the antioxidant enzyme superoxide dismutase (59). On the other hand, ATF4 may regulate inflammation indirectly by inducing ATF3 (60), an ATF4 target gene with prominent anti-inflammatory functions. Acting as a transcriptional repressor, ATF3 forms a homodimer, binds to the promoter regions in NF-κB (35) and IFN-β (61) genes, and represses their transcription. Under several pathological conditions, IFN-α/β may exert pro-inflammatory functions (62, 63). ATF3 can also bind to other transcription factors such as AP-1 (35) and NF-κB (64) to inhibit the expression of pro-inflammatory cytokines downstream of TLR4 signaling. In human monocytes, ATF3 was strongly induced by oxidative stress, while silencing the ATF3 gene increased IL-6 production (65). Taken together, it is suggested that how ATF4 influences inflammation varies and is highly context-dependent, similar to the dichotomous roles of ATF4 in regulating cell death and survival (66).

## 7 Concluding remarks

Given the universal expression and function of eIF2α, ISR is thought to have fundamental roles in maintaining cell viability under various conditions when intracellular and/or extracellular homeostasis is disrupted. However, it is noted that depending on the nature of the stress stimuli, its duration and severity, the extent of eIF2α phosphorylation, and the levels of expression of ATF4 and related transcription factors, ISR can also signal toward cell death (3). Moreover, the roles of ISR induction under pathological conditions may vary in a cell- and context-specific manner. For example, in neuronal cells, ISR signaling is involved in both physiological regulations (such as nervous system development and memory consolidation) and pathogenesis of various neurodegenerative diseases (67, 68). In recent years, ISR has been recognized as a potential drug target; however, given its dual role in cell fate regulation, extensive research has been undertaken to identify pharmacological agents that either enhance or reduce eIF2 phosphorylation depending on the specific disease condition (2, 10, 17, 46, 69–71).

In immune cells, emerging evidence suggests that activation of ISR may repress inflammatory reactions, at least in a context-dependent manner. This effect can be achieved by p-eIF2α-dependent translational regulation, which limits the production of pro-inflammatory factors. In addition, ISR-induced ATF4 production may contribute to the anti-inflammatory effect by reprogramming the transcriptional activity of a panel of genes involved in inflammation. It is proposed that targeting ISR may offer specific therapeutic benefits by curbing acutely heightened inflammatory responses, which are dependent on increased protein synthesis of cytokines and chemokines. During severe inflammation, multiple cytokines are required to act in concert to sustain high levels of pro-inflammatory signaling. Therefore, targeting individual cytokines is likely to have limited therapeutic efficacy, as demonstrated by the failures of various cytokine inhibitors in patients with sepsis (72, 73). Of note, strategies to enhance ISR are expected to produce broader inhibitory effects on multiple cytokine pathways, providing benefits comparable to those of combined therapy. This property of ISR opens a new avenue to discoveries of novel anti-inflammatory agents that have a distinct biological target from existing drugs.

It is noted that finding the optimal time window for ISR induction is to be crucial for treating inflammation, because appropriate production of anti-inflammatory cytokines is critical for the resolution of inflammation in the later stage. For example, p-eIF2α-mediated translation shutdown in the late phase of sepsis was shown to have deleterious effects on sepsis-induced kidney injury (74). Moreover, unlike acute inflammation, many inflammatory diseases result from chronic, low-grade inflammation, for which ISR activation might not be an appropriate treatment option. Perpetuating ISR may have deleterious effects on normal physiological functions [e.g. worsening neurodegenerative diseases by promoting neuronal cell death (75)]. Currently, a range of small molecule PP1/GADD34 inhibitors and PERK activators have been reported [see (10)]. In addition, novel ISR activators such as ISRAC, which inactivates eIF2B by inducing a conformational switch to the inactive state engaged by p-eIF2α, have also been reported (76). Overall, it will be very interesting to elucidate the pharmacological effects of these compounds on inflammatory reactions in immune cells.

## References

[1] Wek RC Jiang HY Anthony TG . Coping with stress: eIF2 kinases and translational control. Biochem Soc Trans. (2006) 34:7–11. doi: 10.1042/bst20060007, PMID: 16246168

[2] Costa-Mattioli M Walter P . The integrated stress response: From mechanism to disease. Science. (2020) 368:eaat5314. doi: 10.1126/science.aat5314, PMID: 32327570 PMC8997189

[3] Pakos-Zebrucka K Koryga I Mnich K Ljujic M Samali A Gorman AM . The integrated stress response. EMBO Rep. (2016) 17:1374–95. doi: 10.15252/embr.201642195, PMID: 27629041 PMC5048378

[4] Wek RC Anthony TG Staschke KA . Surviving and adapting to stress: translational control and the integrated stress response. Antioxid Redox Signal. (2023) 39:351–73. doi: 10.1089/ars.2022.0123, PMID: 36943285 PMC10443206

[5] Chen QM . The odds of protein translation control under stress. Antioxid Redox Signal. (2024) 40:943–7. doi: 10.1089/ars.2023.0478, PMID: 38573012 PMC11538090

[6] Ryoo HD . The integrated stress response in metabolic adaptation. J Biol Chem. (2024) 300:107151. doi: 10.1016/j.jbc.2024.107151, PMID: 38462161 PMC10998230

[7] Hetz C Chevet E Harding HP . Targeting the unfolded protein response in disease. Nat Rev Drug Discov. (2013) 12:703–19. doi: 10.1038/nrd3976, PMID: 23989796

[8] Pavitt GD Ron D . New insights into translational regulation in the endoplasmic reticulum unfolded protein response. Cold Spring Harb Perspect Biol. (2012) 4:a012278. doi: 10.1101/cshperspect.a012278, PMID: 22535228 PMC3367556

[9] Neill G Masson GR . A stay of execution: ATF4 regulation and potential outcomes for the integrated stress response. Front Mol Neurosci. (2023) 16:1112253. doi: 10.3389/fnmol.2023.1112253, PMID: 36825279 PMC9941348

[10] Marciniak SJ Chambers JE Ron D . Pharmacological targeting of endoplasmic reticulum stress in disease. Nat Rev Drug Discov. (2022) 21:115–40. doi: 10.1038/s41573-021-00320-3, PMID: 34702991

[11] Ceulemans H Bollen M . Functional diversity of protein phosphatase-1, a cellular economizer and reset button. Physiol Rev. (2004) 84:1–39. doi: 10.1152/physrev.00013.2003, PMID: 14715909

[12] Bollen M Peti W Ragusa MJ Beullens M . The extended PP1 toolkit: designed to create specificity. Trends Biochem Sci. (2010) 35:450–8. doi: 10.1016/j.tibs.2010.03.002, PMID: 20399103 PMC3131691

[13] Choy MS Yusoff P Lee IC Newton JC Goh CW Page R . Structural and functional analysis of the GADD34:PP1 eIF2α Phosphatase. Cell Rep. (2015) 11:1885–91. doi: 10.1016/j.celrep.2015.05.043, PMID: 26095357 PMC4489983

[14] Jousse C Oyadomari S Novoa I Lu P Zhang Y Harding HP . Inhibition of a constitutive translation initiation factor 2alpha phosphatase, CReP, promotes survival of stressed cells. J Cell Biol. (2003) 163:767–75. doi: 10.1083/jcb.200308075, PMID: 14638860 PMC2173671

[15] Harding HP Zhang Y Zeng H Novoa I Lu PD Calfon M . An integrated stress response regulates amino acid metabolism and resistance to oxidative stress. Mol Cell. (2003) 11:619–33. doi: 10.1016/s1097-2765(03)00105-9, PMID: 12667446

[16] Lu PD Jousse C Marciniak SJ Zhang Y Novoa I Scheuner D . Cytoprotection by pre-emptive conditional phosphorylation of translation initiation factor 2. EMBO J. (2004) 23:169–79. doi: 10.1038/sj.emboj.7600030, PMID: 14713949 PMC1271668

[17] Boyce M Bryant KF Jousse C Long K Harding HP Scheuner D . A selective inhibitor of eIF2alpha dephosphorylation protects cells from ER stress. Science. (2005) 307:935–9. doi: 10.1126/science.1101902, PMID: 15705855

[18] Rajesh K Krishnamoorthy J Kazimierczak U Tenkerian C Papadakis AI Wang S . Phosphorylation of the translation initiation factor eIF2α at serine 51 determines the cell fate decisions of Akt in response to oxidative stress. Cell Death Dis. (2015) 6:e1591. doi: 10.1038/cddis.2014.554, PMID: 25590801 PMC4669752

[19] Muaddi H Majumder M Peidis P Papadakis AI Holcik M Scheuner D . Phosphorylation of eIF2α at serine 51 is an important determinant of cell survival and adaptation to glucose deficiency. Mol Biol Cell. (2010) 21:3220–31. doi: 10.1091/mbc.E10-01-0023, PMID: 20660158 PMC2938387

[20] Roux PP Topisirovic I . Signaling pathways involved in the regulation of mRNA translation. Mol Cell Biol. (2018) 38:e00070–18. doi: 10.1128/mcb.00070-18, PMID: 29610153 PMC5974435

[21] Naz S Battu S Khan RA Afroz S Giddaluru J Vishwakarma SK . Activation of integrated stress response pathway regulates IL-1β production through posttranscriptional and translational reprogramming in macrophages. Eur J Immunol. (2019) 49:277–89. doi: 10.1002/eji.201847513, PMID: 30578631

[22] Battu S Afroz S Giddaluru J Naz S Huang W Khumukcham SS . Amino acid starvation sensing dampens IL-1β production by activating riboclustering and autophagy. PloS Biol. (2018) 16:e2005317. doi: 10.1371/journal.pbio.2005317, PMID: 29621237 PMC5903674

[23] Hofmann S Kedersha N Anderson P Ivanov P . Molecular mechanisms of stress granule assembly and disassembly. Biochim Biophys Acta Mol Cell Res. (2021) 1868:118876. doi: 10.1016/j.bbamcr.2020.118876, PMID: 33007331 PMC7769147

[24] Humeau J Leduc M Cerrato G Loos F Kepp O Kroemer G . Phosphorylation of eukaryotic initiation factor-2α (eIF2α) in autophagy. Cell Death Dis. (2020) 11:433. doi: 10.1038/s41419-020-2642-6, PMID: 32513922 PMC7280501

[25] Tallóczy Z Jiang W Virgin H Leib DA Scheuner D Kaufman RJ . Regulation of starvation- and virus-induced autophagy by the eIF2alpha kinase signaling pathway. Proc Natl Acad Sci U.S.A. (2002) 99:190–5. doi: 10.1073/pnas.012485299, PMID: 11756670 PMC117537

[26] Wang X Dai C Cheng W Wang J Cui X Pan G . Repressing cytokine storm-like response in macrophages by targeting the eIF2α-integrated stress response pathway. Int Immunopharmacol. (2025) 147:113965. doi: 10.1016/j.intimp.2024.113965, PMID: 39752757

[27] Grootjans J Kaser A Kaufman RJ Blumberg RS . The unfolded protein response in immunity and inflammation. Nat Rev Immunol. (2016) 16:469–84. doi: 10.1038/nri.2016.62, PMID: 27346803 PMC5310224

[28] Chipurupalli S Samavedam U Robinson N . Crosstalk between ER stress, autophagy and inflammation. Front Med (Lausanne). (2021) 8:758311. doi: 10.3389/fmed.2021.758311, PMID: 34805224 PMC8602556

[29] Baccala R Gonzalez-Quintial R Lawson BR Stern ME Kono DH Beutler B . Sensors of the innate immune system: their mode of action. Nat Rev Rheumatol. (2009) 5:448–56. doi: 10.1038/nrrheum.2009.136, PMID: 19597511

[30] Wicherska-Pawłowska K Wróbel T Rybka J . Toll-like receptors (TLRs), NOD-like receptors (NLRs), and RIG-I-like receptors (RLRs) in innate immunity. TLRs, NLRs, and RLRs ligands as immunotherapeutic agents for hematopoietic diseases. Int J Mol Sci. (2021) 22:13397. doi: 10.3390/ijms222413397, PMID: 34948194 PMC8704656

[31] McNab F Mayer-Barber K Sher A Wack A O’Garra A . Type I interferons in infectious disease. Nat Rev Immunol. (2015) 15:87–103. doi: 10.1038/nri3787, PMID: 25614319 PMC7162685

[32] Eiermann N Haneke K Sun Z Stoecklin G Ruggieri A . Dance with the devil: stress granules and signaling in antiviral responses. Viruses. (2020) 12:984. doi: 10.3390/v12090984, PMID: 32899736 PMC7552005

[33] McCormick C Khaperskyy DA . Translation inhibition and stress granules in the antiviral immune response. Nat Rev Immunol. (2017) 17:647–60. doi: 10.1038/nri.2017.63, PMID: 28669985

[34] Brocard M Lu J Hall B Borah K Moller-Levet C Georgana I . Murine norovirus infection results in anti-inflammatory response downstream of amino acid depletion in macrophages. J Virol. (2021) 95:e0113421. doi: 10.1128/JVI.01134-21, PMID: 34346771 PMC8475529

[35] Jadhav K Zhang Y . Activating transcription factor 3 in immune response and metabolic regulation. Liver Res. (2017) 1:96–102. doi: 10.1016/j.livres.2017.08.001, PMID: 29242753 PMC5724780

[36] Li D Zhang H Zhong Y . Hepatic GDF15 is regulated by CHOP of the unfolded protein response and alleviates NAFLD progression in obese mice. Biochem Biophys Res Commun. (2018) 498:388–94. doi: 10.1016/j.bbrc.2017.08.096, PMID: 28847729

[37] Dai C Zhang H Zheng Z Li CG Ma M Gao H . Identification of a distinct cluster of GDF15(high) macrophages induced by *in vitro* differentiation exhibiting anti-inflammatory activities. Front Immunol. (2024) 15:1309739. doi: 10.3389/fimmu.2024.1309739, PMID: 38655264 PMC11036887

[38] Asada N Ginsberg P Paust HJ Song N Riedel JH Turner JE . The integrated stress response pathway controls cytokine production in tissue-resident memory CD4(+) T cells. Nat Immunol. (2025) 26:557–66. doi: 10.1038/s41590-025-02105-x, PMID: 40050432 PMC11957990

[39] Robert F Kapp LD Khan SN Acker MG Kolitz S Kazemi S . Initiation of protein synthesis by hepatitis C virus is refractory to reduced eIF2.GTP.Met-tRNA(i)(Met) ternary complex availability. Mol Biol Cell. (2006) 17:4632–44. doi: 10.1091/mbc.e06-06-0478, PMID: 16928960 PMC1635388

[40] Sundrud MS Koralov SB Feuerer M Calado DP Kozhaya AE Rhule-Smith A . Halofuginone inhibits TH17 cell differentiation by activating the amino acid starvation response. Science. (2009) 324:1334–8. doi: 10.1126/science.1172638, PMID: 19498172 PMC2803727

[41] Keller TL Zocco D Sundrud MS Hendrick M Edenius M Yum J . Halofuginone and other febrifugine derivatives inhibit prolyl-tRNA synthetase. Nat Chem Biol. (2012) 8:311–7. doi: 10.1038/nchembio.790, PMID: 22327401 PMC3281520

[42] Pitera AP Szaruga M Peak-Chew SY Wingett SW Bertolotti A . Cellular responses to halofuginone reveal a vulnerability of the GCN2 branch of the integrated stress response. EMBO J. (2022) 41:e109985. doi: 10.15252/embj.2021109985, PMID: 35466425 PMC9156968

[43] Shen Y Wang Y Fu Z Ma Q Song Y Fang L . UPR attenuates the proinflammatory effect of HPDLF on macrophage polarization. Cell Stress Chaperones. (2021) 26:937–44. doi: 10.1007/s12192-021-01234-0, PMID: 34495492 PMC8578276

[44] Wei Y Meng M Tian Z Xie F Yin Q Dai C . Pharmacological preconditioning with the cellular stress inducer thapsigargin protects against experimental sepsis. Pharmacol Res. (2019) 141:114–22. doi: 10.1016/j.phrs.2018.12.017, PMID: 30579975

[45] Payea MJ Dar SA Anerillas C Martindale JL Belair C Munk R . Senescence suppresses the integrated stress response and activates a stress-remodeled secretory phenotype. Mol Cell. (2024) 84:4454–69. doi: 10.1016/j.molcel.2024.10.003, PMID: 39481386 PMC11585442

[46] Krzyzosiak A Sigurdardottir A Luh L Carrara M Das I Schneider K . Target-based discovery of an inhibitor of the regulatory phosphatase PPP1R15B. Cell. (2018) 174:1216–28.e19. doi: 10.1016/j.cell.2018.06.030, PMID: 30057111 PMC6108835

[47] Ravindran R Loebbermann J Nakaya HI Khan N Ma H Gama L . The amino acid sensor GCN2 controls gut inflammation by inhibiting inflammasome activation. Nature. (2016) 531:523–7. doi: 10.1038/nature17186, PMID: 26982722 PMC4854628

[48] Blériot C Dalmas É Ginhoux F Venteclef N . Inflammatory and immune etiology of type 2 diabetes. Trends Immunol. (2023) 44:101–9. doi: 10.1016/j.it.2022.12.004, PMID: 36604203

[49] Donath MY Shoelson SE . Type 2 diabetes as an inflammatory disease. Nat Rev Immunol. (2011) 11:98–107. doi: 10.1038/nri2925, PMID: 21233852

[50] Rai S Szaruga M Pitera AP Bertolotti A . Integrated stress response activator halofuginone protects mice from diabetes-like phenotypes. J Cell Biol. (2024) 223:e202405175. doi: 10.1083/jcb.202405175, PMID: 39150520 PMC11329777

[51] Bonnet MC Weil R Dam E Hovanessian AG Meurs EF . PKR stimulates NF-kappaB irrespective of its kinase function by interacting with the IkappaB kinase complex. Mol Cell Biol. (2000) 20:4532–42. doi: 10.1128/mcb.20.13.4532-4542.2000, PMID: 10848580 PMC85837

[52] Jiang HY Wek SA McGrath BC Scheuner D Kaufman RJ Cavener DR . Phosphorylation of the alpha subunit of eukaryotic initiation factor 2 is required for activation of NF-kappaB in response to diverse cellular stresses. Mol Cell Biol. (2003) 23:5651–63. doi: 10.1128/mcb.23.16.5651-5663.2003, PMID: 12897138 PMC166326

[53] Deng J Lu PD Zhang Y Scheuner D Kaufman RJ Sonenberg N . Translational repression mediates activation of nuclear factor kappa B by phosphorylated translation initiation factor 2. Mol Cell Biol. (2004) 24:10161–8. doi: 10.1128/mcb.24.23.10161-10168.2004, PMID: 15542827 PMC529034

[54] Abdel-Nour M Carneiro LAM Downey J Tsalikis J Outlioua A Prescott D . The heme-regulated inhibitor is a cytosolic sensor of protein misfolding that controls innate immune signaling. Science. (2019) 365:eaaw4144. doi: 10.1126/science.aaw4144, PMID: 31273097 PMC10433729

[55] Almeida-da-Silva CLC Savio LEB Coutinho-Silva R Ojcius DM . The role of NOD-like receptors in innate immunity. Front Immunol. (2023) 14:1122586. doi: 10.3389/fimmu.2023.1122586, PMID: 37006312 PMC10050748

[56] Allagnat F Fukaya M Nogueira TC Delaroche D Welsh N Marselli L . C/EBP homologous protein contributes to cytokine-induced pro-inflammatory responses and apoptosis in β-cells. Cell Death Differ. (2012) 19:1836–46. doi: 10.1038/cdd.2012.67, PMID: 22653339 PMC3469067

[57] Khelfi A . Oxidative stress in inflammation. In: Andreescu S Henkel R & Khelfi A , editors. Biomarkers of Oxidative Stress. Switzerland: Springer Nature (2024). p. 13–43.

[58] Kreß JKC Jessen C Hufnagel A Schmitz W Xavier da Silva TN Ferreira Dos Santos A . The integrated stress response effector ATF4 is an obligatory metabolic activator of NRF2. Cell Rep. (2023) 42:112724. doi: 10.1016/j.celrep.2023.112724, PMID: 37410595

[59] Fusakio ME Willy JA Wang Y Mirek ET Al Baghdadi RJ Adams CM . Transcription factor ATF4 directs basal and stress-induced gene expression in the unfolded protein response and cholesterol metabolism in the liver. Mol Biol Cell. (2016) 27:1536–51. doi: 10.1091/mbc.E16-01-0039, PMID: 26960794 PMC4850040

[60] Kilberg MS Shan J Su N . ATF4-dependent transcription mediates signaling of amino acid limitation. Trends Endocrinol Metab. (2009) 20:436–43. doi: 10.1016/j.tem.2009.05.008, PMID: 19800252 PMC3587693

[61] Labzin LI Schmidt SV Masters SL Beyer M Krebs W Klee K . ATF3 is a key regulator of macrophage IFN responses. J Immunol. (2015) 195:4446–55. doi: 10.4049/jimmunol.1500204, PMID: 26416280

[62] López de Padilla CM Niewold TB . The type I interferons: Basic concepts and clinical relevance in immune-mediated inflammatory diseases. Gene. (2016) 576:14–21. doi: 10.1016/j.gene.2015.09.058, PMID: 26410416 PMC4666791

[63] Crow MK Ronnblom L . Type I interferons in host defence and inflammatory diseases. Lupus Sci Med. (2019) 6:e000336. doi: 10.1136/lupus-2019-000336, PMID: 31205729 PMC6541752

[64] Kwon JW Kwon HK Shin HJ Choi YM Anwar MA Choi S . Activating transcription factor 3 represses inflammatory responses by binding to the p65 subunit of NF-κB. Sci Rep. (2015) 5:14470. doi: 10.1038/srep14470, PMID: 26412238 PMC4585983

[65] Hoetzenecker W Echtenacher B Guenova E Hoetzenecker K Woelbing F Brück J . ROS-induced ATF3 causes susceptibility to secondary infections during sepsis-associated immunosuppression. Nat Med. (2011) 18:128–34. doi: 10.1038/nm.2557, PMID: 22179317 PMC3555699

[66] Pitale PM Gorbatyuk O Gorbatyuk M . Neurodegeneration: keeping ATF4 on a tight leash. Front Cell Neurosci. (2017) 11:410. doi: 10.3389/fncel.2017.00410, PMID: 29326555 PMC5736573

[67] Bellato HM Hajj GN . Translational control by eIF2α in neurons: Beyond the stress response. Cytoskeleton (Hoboken). (2016) 73:551–65. doi: 10.1002/cm.21294, PMID: 26994324

[68] Bravo-Jimenez MA Sharma S Karimi-Abdolrezaee S . The integrated stress response in neurodegenerative diseases. Mol Neurodegener. (2025) 20:20. doi: 10.1186/s13024-025-00811-6, PMID: 39972469 PMC11837473

[69] Luh LM Bertolotti A . Potential benefit of manipulating protein quality control systems in neurodegenerative diseases. Curr Opin Neurobiol. (2020) 61:125–32. doi: 10.1016/j.conb.2020.02.009, PMID: 32199101

[70] Tsaytler P Harding HP Ron D Bertolotti A . Selective inhibition of a regulatory subunit of protein phosphatase 1 restores proteostasis. Science. (2011) 332:91–4. doi: 10.1126/science.1201396, PMID: 21385720

[71] Das I Krzyzosiak A Schneider K Wrabetz L D’Antonio M Barry N . Preventing proteostasis diseases by selective inhibition of a phosphatase regulatory subunit. Science. (2015) 348:239–42. doi: 10.1126/science.aaa4484, PMID: 25859045 PMC4490275

[72] Chousterman BG Swirski FK Weber GF . Cytokine storm and sepsis disease pathogenesis. Semin Immunopathol. (2017) 39:517–28. doi: 10.1007/s00281-017-0639-8, PMID: 28555385

[73] Hattori Y Hattori K Suzuki T Matsuda N . Recent advances in the pathophysiology and molecular basis of sepsis-associated organ dysfunction: Novel therapeutic implications and challenges. Pharmacol Ther. (2017) 177:56–66. doi: 10.1016/j.pharmthera.2017.02.040, PMID: 28232275

[74] Hato T Maier B Syed F Myslinski J Zollman A Plotkin Z . Bacterial sepsis triggers an antiviral response that causes translation shutdown. J Clin Invest. (2019) 129:296–309. doi: 10.1172/jci123284, PMID: 30507610 PMC6307966

[75] Bond S Lopez-Lloreda C Gannon PJ Akay-Espinoza C Jordan-Sciutto KL . The integrated stress response and phosphorylated eukaryotic initiation factor 2α in neurodegeneration. J Neuropathol Exp Neurol. (2020) 79:123–43. doi: 10.1093/jnen/nlz129, PMID: 31913484 PMC6970450

[76] Shilliday F Gancedo-Rodrigo M George G Adhikari S Ashraf SN Barrey EJC . A molecular stabiliser of an inhibitory eIF2B-eIF2(αP) complex activates the Integrated Stress Response. bioRxiv. (2025). doi: 10.1101/2025.09.25.678332. Preprint. PMC13358149 42091608

